# Rho‐kinase inhibitor Y‐27632 facilitates the proliferation, migration and pluripotency of human periodontal ligament stem cells

**DOI:** 10.1111/jcmm.13222

**Published:** 2017-06-29

**Authors:** Ting Wang, Wenyan Kang, Lingqian Du, Shaohua Ge

**Affiliations:** ^1^ Shandong Provincial Key Laboratory of Oral Tissue Regeneration School of Stomatology Shandong University Jinan China; ^2^ Department of Periodontology School of Stomatology Shandong University Jinan China; ^3^ Department of Stomatology The Second Hospital of Shandong University Jinan China

**Keywords:** periodontal ligament stem cells, Y‐27632, ROCK inhibitor, proliferation, chemotaxis, differentiation

## Abstract

The selective *in vitro* expansion and differentiation of multipotent stem cells are critical steps in cell‐based regenerative therapies, while technical challenges have limited cell yield and thus affected the success of these potential treatments. The Rho GTPases and downstream Rho kinases are central regulators of cytoskeletal dynamics during cell cycle and determine the balance between stem cells self‐renewal, lineage commitment and apoptosis. Trans‐4‐[(1R)‐aminoethyl]‐N‐(4‐pyridinyl)cylohexanecarboxamidedihydrochloride (Y‐27632), Rho‐associated kinase (ROCK) inhibitor, involves various cellular functions that include actin cytoskeleton organization, cell adhesion, cell motility and anti‐apoptosis. Here, human periodontal ligament stem cells (PDLSCs) were isolated by limiting dilution method. Cell counting kit‐8 (CCK8), 5‐ethynyl‐2′‐deoxyuridine (EdU) labelling assay, cell apoptosis assay, cell migration assay, wound‐healing assay, alkaline phosphatase (ALP) activity assay, Alizarin Red S staining, Oil Red O staining, quantitative real‐time polymerase chain reaction (qRT‐PCR) were used to determine the effects of Y‐27632 on the proliferation, apoptosis, migration, stemness, osteogenic and adipogenic differentiation of PDLSCs. Afterwards, Western blot analysis was performed to elucidate the mechanism of cell proliferation. The results indicated that Y‐27632 significantly promoted cell proliferation, chemotaxis, wound healing, fat droplets formation and pluripotency, while inhibited ALP activity and mineral deposition. Furthermore, Y‐27632 induced PDLSCs proliferation through extracellular‐signal‐regulated kinase (ERK) signalling cascade. Therefore, control of Rho‐kinase activity may enhance the efficiency of stem cell‐based treatments for periodontal diseases and the strategy may have the potential to promote periodontal tissue regeneration by facilitating the chemotaxis of PDLSCs to the injured site, and then enhancing the proliferation of these cells and maintaining their pluripotency.

## Introduction

Multipotent stem cells within periodontal ligament (PDL), termed PDLSCs, have been isolated as ideal cellular sources for periodontal tissue repair and regeneration 1. PDLSCs exhibit osteogenic, adipogenic and chondrogenic characteristics under inductive culture conditions *in vitro* 2, 3. Furthermore, PDLSCs transplantation therapies have the potential to promote the formation of new bone, new cementum and functional PDL in damaged periodontium in animal models 4, 5, 6, 7. Despite this potential, damaged periodontium contains relatively few stem cells, and obtaining sufficient number of stem cells for transplantation requires efficient isolation and *in vitro* expansion. Furthermore, stem cells must migrate to the defect area and differentiate into the appropriate functional phenotype to participate in wound healing, tissue repair and regeneration. The molecular and cellular mechanisms underneath are complex and remain poorly understood.

Accumulating evidence suggests that the Rho GTPases and downstream effectors such as Rho kinases regulate cell proliferation, adhesion, migration and apoptosis by affecting cell shape, cytoskeletal dynamics and cell‐cell contacts 8, 9. Moreover, the Rho‐kinase inhibitor appears to enhance the survival of stem cells from a variety of sources, thereby improve the yield of differentiated cells 10, and inhibit the apoptosis of stem cells‐derived neuronal progenitors following animal transplantation 11.

Trans‐4‐[(1R)‐aminoethyl]‐N‐(4‐pyridinyl)cylohexanecarboxamidedihydrochloride (Y‐27632) is a dihydrochloride cell‐permeable small molecule and contains a 4‐aminopyridine ring. The chemical formula is C_14_H_23_Cl_2_N_3_O, and the chemical structure formula is shown in Figure S1. In addition, as a pyrimidine derivative, Y‐27632 specifically inhibits ROCK and involves in various cellular functions that include actin cytoskeleton organization, cell adhesion, cell motility and anti‐apoptosis through binding to the Rho‐kinase adenosine triphosphate (ATP) binding pocket in an ATP‐competitive manner 12. Zhang and coworkers reported that Y‐27632 increased cloning efficiency of murine prostate basal epithelial cells. The increased cloning efficiency was due to the suppression of the dissociation‐induced RhoA/ROCK activation‐mediated apoptosis of prostate stem cells 13. Y‐27632 was a potentially powerful reagent that was able to enhance the proliferation of cultured bovine corneal endothelial cells (B‐CECs) and significantly enhanced the adhesion and migration of B‐CECs. B‐CECs treated with Y‐27632 exhibited more vigorous growth and more spread morphology 14.

The aforementioned observations raise a possibility that in addition to the known growth‐factor‐mediated pathway, Y‐27632 may serve as an alternative regulator of PDLSCs bioactivity. To our knowledge, only one study investigated the regulation of osteogenic differentiation of PDL cells by Y‐27632 15, and no research evaluated the effects of Y‐27632 on the biobehaviour of PDLSCs. Therefore, the purpose of this study was to explore the effects of Y‐27632 on the proliferation, apoptosis, migration, wound‐healing ability, differentiation capacities and stemness maintenance of PDLSCs, and to evaluate whether Y‐27632 could be an appropriate reagent in regenerative dentistry.

## Materials and methods

### Ethical statements

The study protocol was approved by the Medical Ethical Committee of School of Stomatology, Shandong University (Protocol Number: GR201603) and written informed consent was obtained from every individual participant. All the protocols were carried out in accordance with the guidelines of the National Institute of Health (NIH).

### Cultivation of human PDLSCs

Human PDLSCs were harvested from healthy PDL of the teeth extracted for orthodontic reasons. The teeth were stored in Dulbecco's modified Eagle's medium (DMEM; Hyclone, Logan, UT, USA) with penicillin G (100 U/ml) and streptomycin (100 mg/ml). Human PDL tissue was scraped from the middle part of the root surface as we previously described 16, 17. The PDL tissue was cut into some small pieces and then digested with collagenase I (3 mg/ml, Sigma‐Aldrich, St. Louis, MO, USA) and dispase II (4 mg/ml; Sigma‐Aldrich) for 40 min. To further isolate and purify the stem cells, single‐cell suspensions of primary cells were cloned with the limiting‐dilution method as previously described 18. After digestion, the single‐cell suspension was filtered through a 70 μm strainer. Half of the single‐cell suspensions were plated at a concentration of 60 cells/cm^2^ in 10‐cm tissue culture dishes for the selection of single cell‐derived colonies in DMEM supplemented with 10% foetal bovine serum (FBS; BioInd, Kibbutz, Israel), 50 mg/ml streptomycin and 50 U/ml penicillin G in a humidified atmosphere (37°C, 5% CO_2_). The non‐adherent cells were removed 3 days later and the basic medium was changed three times per week. Individual plastic‐adherent, MSC‐like colonies grown for 10–14 days in 10‐cm tissue culture dishes were isolated using colony rings and expanded using individual tissue culture flask. Single colonies (50 or more cells clusters) of primary cells were isolated used colony rings. Colony ring was made by cutting down one part of the 1000 μl pipette tip with the blade on fire. The ring was put into a big class plate with wax on the base and made autoclaved. We circled location of the selected single colony under microscope (Olympus, Tokyo, Japan) and put the ring around the colony. Cell clusters from the colony were then detached with 0.05% trypsin/EDTA and transferred into 96‐well plates. Cells were then transferred in sequence from the 96‐well plates to a 48‐well, a 24‐well, a 6‐well plates until it was possible to continue cultivation in flasks.

### Cell proliferation assay

The effect of Y‐27632 on PDLSCs proliferation was assayed by CCK8 (Dojindo Laboratories, Kumamoto, Japan) and 5‐ethynyl‐2′‐deoxyuridine (EdU) labelling assay according to the manufacturer's instructions. In brief, for CCK8 assay, cultured PDLSCs were suspended in culture medium with 0.1% FBS and inoculated in a 96‐well plate (5 **×** 10^3^ cells/well) along with 0, 5, 10, 20, 40 μM Y‐27632 (Gene Operation, Ann Arbor, MI, USA). After 48 hrs incubation, 10 μl CCK8 solution was added to each well. The plate was incubated for additional 2.5 hrs before measuring the absorbance at 450 nm wavelength using a microplate reader (SPECTROstar Nano; BMG Labtech, Ortenberg, Germany). For EdU labelling assay, PDLSCs were seeded on the glass coverslips at 2 × 10^4^ cells per well in 24‐well plates and incubated in DMEM containing 10% FBS with or without 5, 10, 20, 40 μM Y‐27632 for 24 hrs. EdU (Ribobio, Guangzhou, China) was added to each well for 2 hrs, and the cells were fixed and stained by 1 × Apollo567 for 30 min. without light. Cells were counterstained with 4′,6‐diamidino‐2‐phenylindole (DAPI) (nuclear staining). The stained cells were examined with fluorescence microscope (Olympus) and photographed with camera. The proliferation rate of cells was assessed with the proportion of EdU‐positive nucleus (red) to blue fluorescent nucleus by counting six microscopic fields randomly for each well in five separate experiments.

### Colony forming efficiency assay

PDLSCs were inoculated in six‐well plates at a seeding density of 1 × 10^3^ cells/well and stimulated with 0, 10, 20 μM Y‐27632. After 10 days, the cells were fixed in 4% paraformaldehyde for 30 min. Total number of cell colonies was photographed and counted after staining with crystal violet (Solarbio, Beijing, China) for 5 min. The ability to form colonies (groups of 50 or more adhering cells derived from the same mother cell) was assessed.

### Analysis of apoptosis by flow cytometry

PDLSCs were cultured in 6‐cm Petri dishes. After serum starvation for 24 hrs, cells were treated with 0, 10, 20 μM Y‐27632 for 48 hrs without serum, and cells cultured with 10% FBS was served as positive control. Afterwards, cells were trypsinized. The resuspended cells were washed with PBS and stained with annexin V‐FITC and propidium iodide (PI) according to the instructions of AnnexinV‐FITC apoptosis detection kit (Wanleibio, Shenyang, China). Cells were then analysed by flow cytometry (CytoFLEX; Beckman Coulter, Brea, CA, USA).

### Cell migration and wound‐healing assays

The migratory effect of Y‐27632 on human PDLSCs was evaluated in transwell chamber (Corning, NY, USA) with an 8 μm pore size polycarbonate membrane in 24‐well plates. In brief, 1 × 10^5^ cells in 200 μl DMEM containing 0.1% FBS were cultured in the upper chamber, and the lower plates were supplemented with 10 or 20 μM Y‐27632 in 500 μl DMEM containing 0.1% FBS. Medium with 0.1% FBS served as a negative control and medium containing 10% FBS served as a positive control. The chambers were incubated for 20 hrs. After removal of non‐migrated cells on the upper layer of the membrane, cells that had migrated through the membrane were fixed in 4% paraformaldehyde, stained with 0.1% crystal violet and counted in six randomly selected high power microscopic fields (×100) per filter by blind evaluation.

In wound‐healing assay, PDLSCs were inoculated in six‐well culture plates until the confluence reached 90%. After serum starvation for 24 hrs, a sterile pipette tip was used to scratch the monolayer. Then medium containing 0, 20 μM Y‐27632 with 0.1% FBS was added to each plate. The distance that cells had migrated was photographed by a digital camera under Inverted microscope (Olympus) at the same position at 0, 6, 12 and 24 hrs for later calculation. Image Pro Plus 6.0 software (Media Cybernetics, Bethesda, MD, USA) was used to measure and calculate the distance that the cells had migrated.

### ALP activity assay

PDLSCs were seeded in 6‐well plates at a density of 2 × l0^5^ cells/well in osteogenic inductive medium (DMEM supplemented with 10% FBS, 10^−8^M dexamethasone, 50 mg/l ascorbic acid and 10 mM β‐glycerophosphate) with 0, 10, 20 μM Y‐27632. After 7 and 14 days of incubation, cells were scraped into 1% TritonX‐100 (Solarbio). Afterwards, cells were sonicated and centrifuged at 12,000 *g* for 10 min. BCA Protein Assay Kit (KeyGen, Nanjing, China) was used to measure the concentration of protein. ALP activity was assayed according to the instruction of ALP activity assay kit (Nanjing Jiancheng Bioengineering Institute, Nanjing, China) and the absorbance was measured at 520 nm wavelength with a microplate reader (SPECTROstar Nano; BMG Labtech).

### Alizarin Red S staining

PDLSCs were seeded in 6‐well plates at a density of 2 × l0^5^ cells/well and were cultured with osteogenic inductive medium with or without 10 or 20 μM Y‐27632. After 28 days induction, cells were fixed in 4% paraformaldehyde, extracellular matrix calcification was examined with 2% Alizarin Red S (PH 4.2; Sigma‐Aldrich) for 15 min. For quantifying the relative amount of calcium, 400 μl 10% (w/v) cetylpyridinium chloride (CPC; Sigma‐Aldrich) and 10 mM sodium phosphate solution were added to the stained dishes and the absorbance of extracted dye was determined at 562 nm wavelength.

### Oil Red O staining

PDLSCs were seeded in 6‐well plates at a density of 2 × l0^5^ cells per well and were cultured with adipogenic inductive medium (DMEM supplemented with 10% FBS, 1 μM dexamethasone, 10 μg/ml insulin, 0.5 mM 3‐isobutyl‐1‐methylxanthine and 0.2 mM indomethacin) with or without the presence of 10 or 20 μM Y‐27632. After 28 days induction, Oil Red O (Solarbio) staining was performed to detect oil deposition. Then 700‐μl isopropyl alcohol was added to the stained dishes and the absorbance of extracted dye was determined at 510 nm.

### qRT‐PCR

Total RNA of cells with different treatments was extracted with Trizol^®^ (Takara, Tokyo, Japan), mRNA was reverse‐transcribed to cDNA using PrimeScript™ RT reagent Kit with gDNA Eraser (Takara). qRT‐PCR reaction was performed with SYBR^®^ Premix Ex Taq II (Tli RNaseH Plus; Takara) on Light Cycler Roche 480 II Real‐Time PCR System (Roche, Basel Switzerland) in triplicate. The primer sequences were listed in Table 1. GAPDH primers were used to normalize the samples. Data were analysed using the 2^−(ΔCt)^ method.

**Table 1:** Primer sequences for qRT‐PCR

Gene	Forward primer (5′–3′)	Reverse primer (5′–3′)
GAPDH	GCACCGTCAAGGCTGAGAAC	TGGTGAAGACGCCAGTGGA
ALP	ATGGGATGGGTGTCTCCACA	CCACGAAGGGGAACTTGTC
Runx2	TCCACACCATTAGGGACCATC	TGCTAATGCTTCGTGTTTCCA
PPARγ	CTCCTATTGACCCAGAAAGC	GTAGAGCTGAGTCTTCTCAG
LPL	AGGACACTTGCCACCTCATTC	ACAGCCAGTCCACCACAATG
c‐Myc	CCCGCTTCTCTGAAAGGCTCTC	CTCTGCTGCTGCTGCTGGTAG
Nanog	CAGAAGGCCTCACACCTAC	ATTGTTCCAGGTCTGGTTGC
Klf4	CAGAGGAGCCCAACCAAAGAG	CGGTAGTGCCTGGTCAGTTCATC
Oct4	CACTGTACTCCTCGGTCCCTTTC	CAGGCACCTCAGTTTGAATGC

### Western blot

PDLSCs were washed with ice‐cold PBS and lysed using a RIPA lysis buffer containing 1% PMSF (Solarbio) and 1% phosphatase inhibitor (Boster, Wuhan, China) and then were centrifuged at 12,000 *g* for 10 min. Protein concentration was measured by a BCA Protein Assay Kit. Proteins were added with SDS‐PAGE loading buffer and then denaturated at 100°C for 5 min. 20 μg/lane proteins were loaded to SDS‐PAGE gels and transferred to PVDF membranes (Millipore, Billerica, MA, USA). Membranes were blocked with non‐fat dry milk for 1 hr, blotted primary antibodies overnight at 4°C and subsequently incubated with horseradish peroxidase‐labelled secondary antibodies (1:2000; Beyotime, Shanghai, China) for 1 hr at room temperature. The membrane was washed three times in tris‐buffered saline with 1‰ Tween20 (TBST). The immunoreactive bands were visualized using enhanced chemiluminescence reagents (Millipore). The level of each protein was normalized to GAPDH before statistical analysis. Image J 1.44 software (NIH, Bethesda, Maryland, USA) was used to quantify the protein expression. The primary antibodies used were as follows: rabbit anti‐phospho‐ERK1/2 (1:1000; Cell Signaling Technology, Danvers MA, USA), rabbit anti‐ERK1/2 (1:2000; Cell Signaling Technology), rabbit anti‐phospho‐p38 (1:1000; Cell Signaling Technology) and GAPDH (1:10,000; Proteintech, Chicago, IN, USA).

### Statistical analysis

Data were collected and expressed as the mean ± standard error of the mean (S.E.M.) and were analysed using SPSS 19.0 (IBM, Armonk, NY, USA) with differences between groups assessed by one‐way anova. Statistical probability of *P* < 0.05 was considered significant.

## Results

### Y‐27632 enhanced the proliferation of PDLSCs

**Figure 1:**
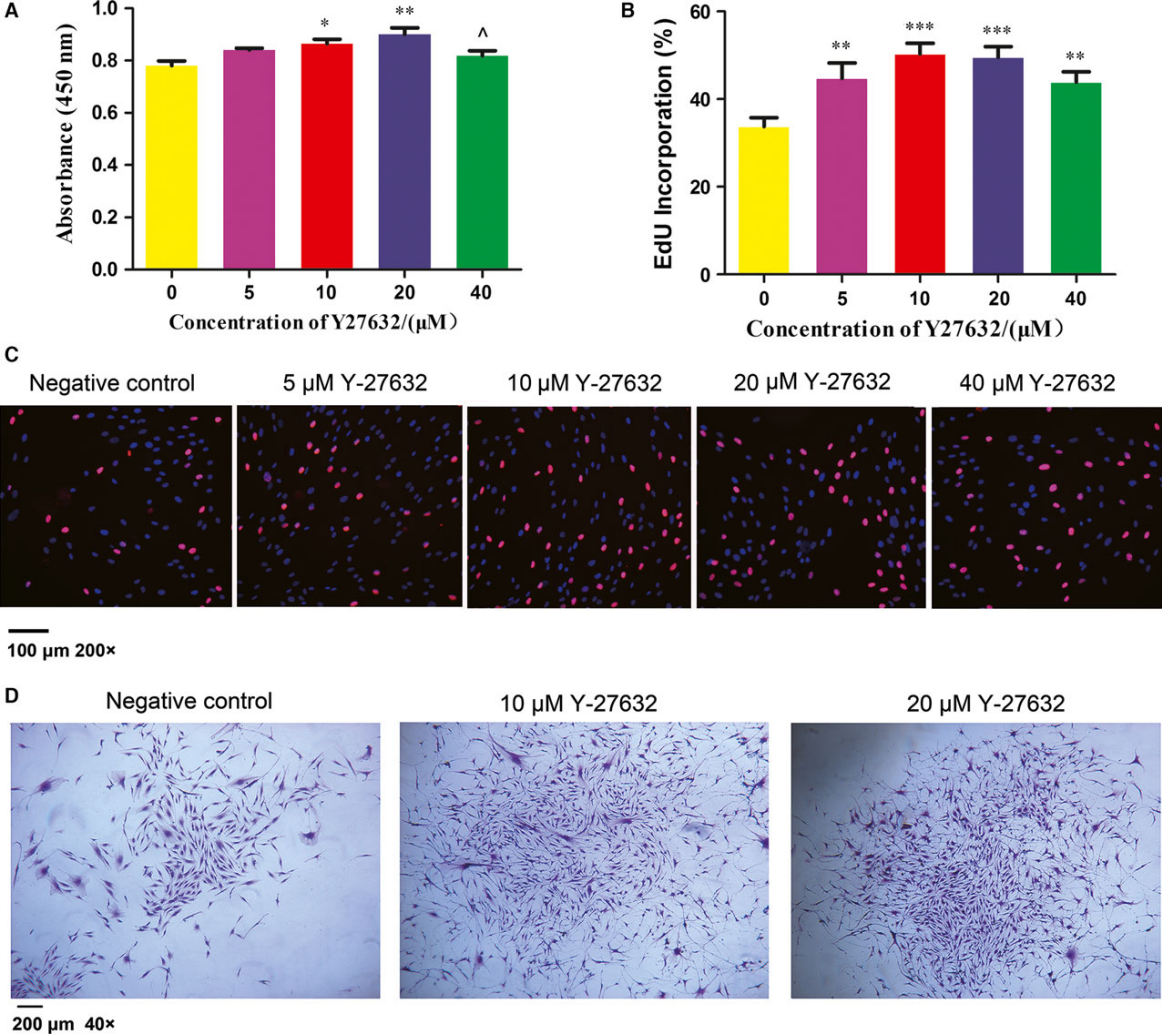
Effects of Y‐27632 on PDLSCs proliferation and colony formation. (**A**) PDLSCs were treated with 0, 5, 10, 20 and 40 μM Y‐27632 for 48 hrs, the number of viable PDLSCs was analysed using CCK8 assay. Data were presented as the mean ± S.E.M.. *P < 0.05, **P < 0.01 compared with negative control; ^*P* < 0.05 compared with 20 μM group. (**B**) Quantification of EdU‐positive PDLSCs was expressed by the percentage of DAPI‐positive cells, and the data were presented as mean ± S.E.M., **P < 0.01, ***P < 0.001 compared with negative control. (**C**) EdU labelling analysis showed the fluorescence images of PDLSCs stimulated with 0, 5, 10, 20, 40 μM Y‐27632 (EdU, red fluorescent signals, DAPI, blue signals). Scale bar: 100 μm (×200). (**D**) PDLSCs were inoculated with 0, 10, 20 μM Y‐27632. After 10 days, cells were stained and colony‐forming efficiency was counted, scale bar: 200 μm (×40). Data were presented as the mean ± S.E.M. of three independent experiments.

The number of viable PDLSCs in different concentration of Y‐27632 was determined by CCK8 assay. The results showed that the proliferation of PDLSCs treated with 10 and 20 μM Y‐27632 was significantly higher than control group (*P* < 0.05) and cell proliferation peaked at the concentration of 20 μM (*P* < 0.01) after Y‐27632 treatment for 48 hrs (Fig. 1A). However, 40 μM Y‐27632 inhibited cell proliferation compared with 20 μM Y‐27632 (*P* < 0.05). Moreover, EdU labelling assay showed that the ratio of EdU‐positive cells in PDLSCs treated with 5, 10, 20 and 40 μM Y‐27632 (44.70 ± 3.53%, 50.3 ± 2.42%, 49.55 ± 2.43%, 43.85±2.37%, respectively) were remarkably enhanced when compared to the negative control group (33.73±2.06%, *P* < 0.01; Fig. 1B and C) and the optimal concentrations were 10 and 20 μM (*P* < 0.001). Thereby, 10 and 20 μM Y‐27632 significantly promoted the proliferation of PDLSCs and were used for subsequent experiments.

The result of colony‐forming efficiency assay showed that there was no significant difference between control group and experimental groups (38.33 ± 2.52 *versus* 39 ± 3, 40.33 ± 3.51 colonies/well, *P* > 0.05). However, the sizes of single‐cell‐derived colonies in experimental groups were larger than that in negative control group (Fig. 1D), and cells in experimental groups exhibited star‐like morphology.

### Y‐27632 had no significant effect on apoptosis of PDLSCs

**Figure 2:**
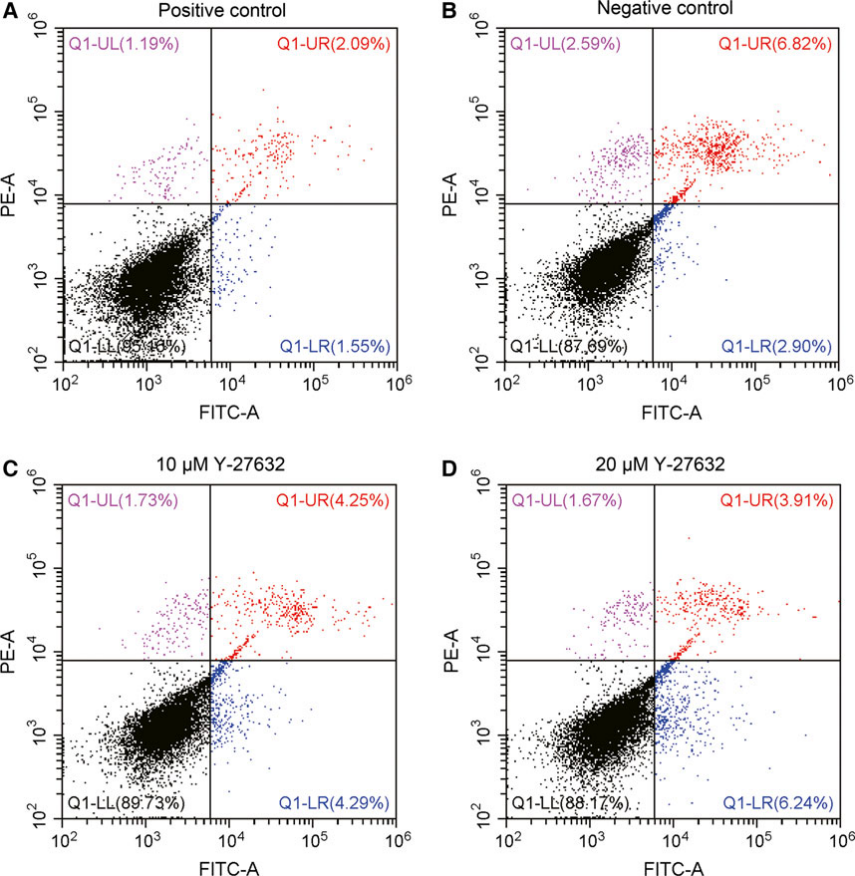
Effect of Y‐27632 on PDLSCs apoptosis. PDLSCs were treated with 10% FBS, 0, 10 and 20 μM Y‐27632 for 48 hrs after serum starvation for 24 hrs, and cell apoptosis was determined by flow cytometry using annexin V‐FITC and propidium iodide (PI) staining. Data were presented as the mean ± S.E.M. of three independent experiments.

The effect of Y‐27632 on PDLSCs apoptosis was analysed using flow cytometry analysis. FITC‐labelled human recombinant annexin V is a phospholipid binding protein (annexin: a marker for early apoptosis) and PI is a nucleic acid dye (PI: a marker for late apoptosis and cell death). The combination of Annexin and PI can distinguish among early apoptosis, late apoptosis and cell death. The rate of cell apoptosis contains early apoptosis and late apoptosis. In our research, the apoptosis rates of positive control (culture medium with 10% FBS) were significantly lower than negative control (culture medium without FBS and Y‐27632), 10 and 20 μM Y‐27632 (3.55 ± 0.11% *versus* 8.91 ± 0.8%, 8.42 ± 0.13%, 10.05 ± 0.19%, *P* < 0.05), while there was no significant difference among 10, 20 μM Y‐27632 and negative control (8.42 ± 0.13%, 10.05 ± 0.19% *versus* 8.91 ± 0.8%, *P* > 0.05; Fig. 2).

### Y‐27632 enhanced migration and wound‐healing capability of PDLSCs

**Figure 3:**
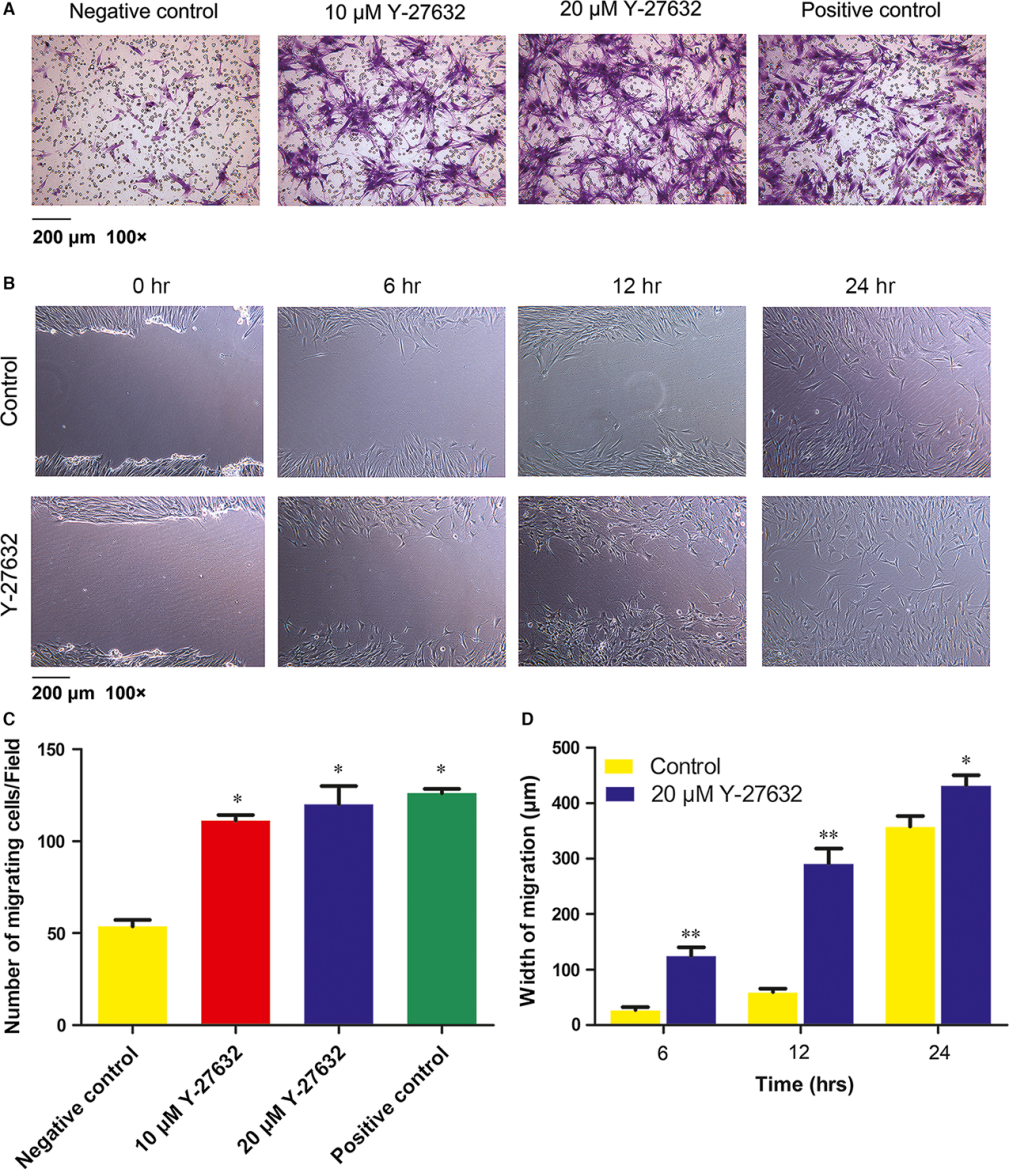
Effects of Y‐27632 on cell migration and wound‐healing capability. (**A, C**) PDLSCs were inoculated in the upper chamber. After 20 hrs incubation with or without Y‐27632, the cells that migrated to the undersurface of the membrane were stained and calculated, scale bar: 200 μm (×100). Data were presented as the mean ± S.E.M. of three independent experiments. *P < 0.05 compared with negative control. (**B, D**) PDLSCs monolayers were scratched and incubated with or without 20 μM Y‐27632 for 24 hrs. The average wound widths were analysed using Image Pro Plus 6.0 software and was expressed relative to 0 hr. Scale bar 200 μm (×100). Data were presented as the mean ± S.E.M. of three independent experiments. *P < 0.05, **P < 0.01 compared with negative control.

The chemotactic capability of Y‐27632 on PDLSCs was quantified with transwell migration assay. Y‐27632 at 10 and 20 μM significantly enhanced PDLSCs migration compared with negative control group (111.1 ± 4.9, 120.03 ± 11.96 *versus* 53.86 ± 5.93 cells/field, *P* < 0.05; Fig. 3A and C). However, there was no significant difference between two dosages of Y‐27632 groups and positive control group (111.1 ± 4.9, 120.03 ± 11.96 *versus* 126.02 ± 4.74 cells/field, *P* > 0.05).

Next, we tested the width of cell migration in wound‐healing assay with the treatment of 20 μM Y‐27632 (Fig. 3B and D). The result indicated that the migration width of experimental group with Y‐27632 treatment was significantly larger than control group after 6, 12 and 24 hrs (*P* < 0.05).

### Y‐27632 inhibited ALP activity, formation of mineral deposition and osteogenic differentiation of PDLSCs

**Figure 4:**
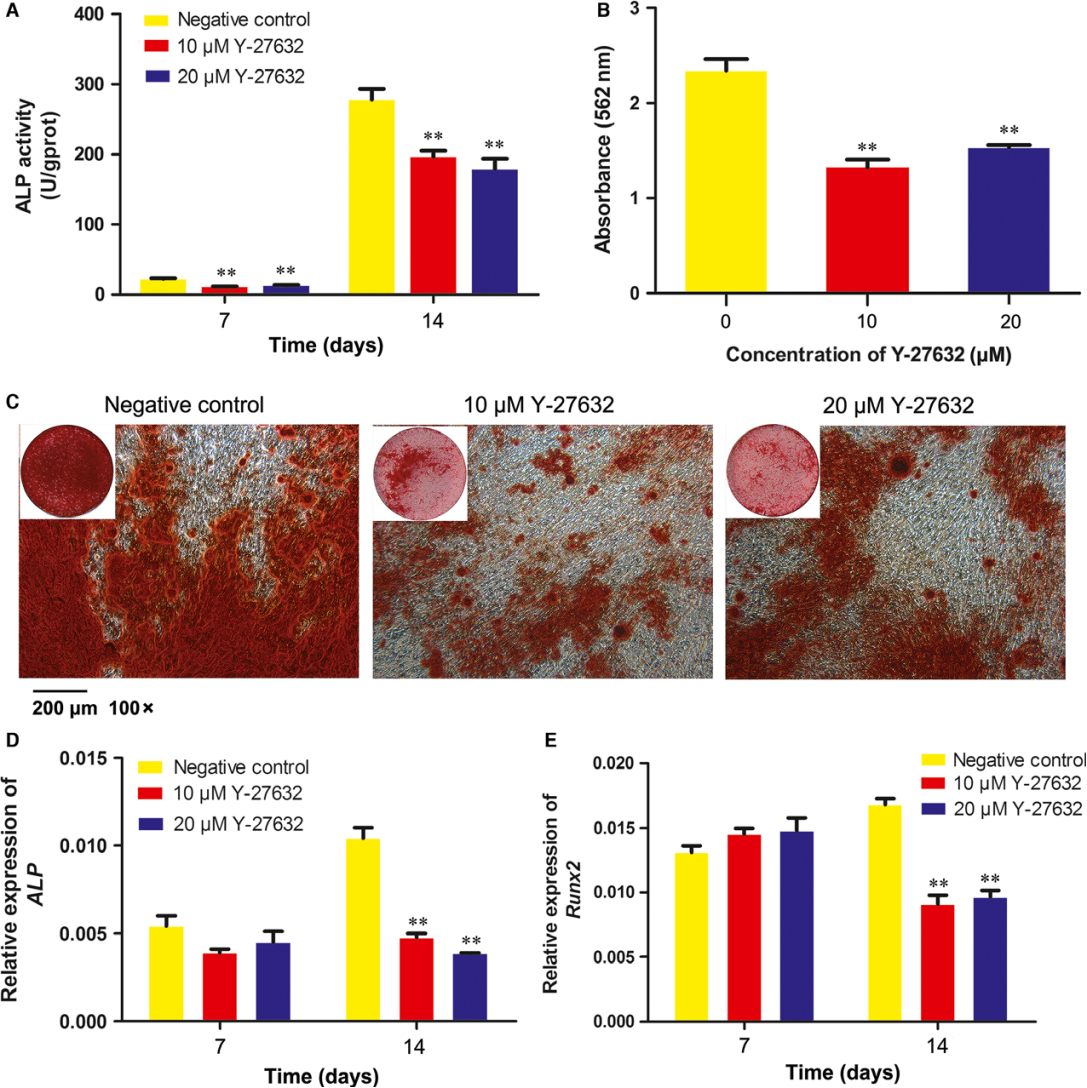
Effect of Y‐27632 on osteogenic differentiation. (**A**) PDLSCs were cultured in osteogenic medium with or without Y‐27632 for 7 and 14 days. ALP activity was measured. (**B, C**) PDLSCs were cultured in osteogenic medium for 28 days, and Alizarin Red staining was performed, scale bar:200 μm (×100). 10% CPC was added and the concentration of calcium deposition was quantified by absorbance at 562 nm. (**D, E**) PDLSCs were cultured in osteogenic medium for 7 and 14 days and the expression of osteogenesis‐related genes were analysed by qRT‐PCR. Data were presented as the mean ± S.E.M. of three independent experiments. **P < 0.01 compared with negative control group.

ALP activity has been widely used as a marker in the early osteogenic differentiation of stem cells. In our research, ALP activity was measured after 7, 14 days with or without Y‐27632 treatment (Fig. 4A). At day 7, 10 and 20 μM Y‐27632 significantly inhibited ALP activity compared with negative control group (*P* < 0.01). At day 14, ALP activity increased in all three groups while ALP activity was significantly down‐regulated in 10 and 20 μM Y‐27632 groups than that in control group (*P* < 0.01).

Extracellular matrix calcification was determined by Alizarin Red S staining (Fig. 4C) and the relative amount of calcium was quantified (Fig. 4B). The application of 10 and 20 μM Y‐27632 significantly inhibited the mineralization in comparison with control group (*P* < 0.01).

Moreover, the effect of Y‐27632 on osteogenic differentiation of PDLSCs was determined by evaluating the gene expression of *ALP* and *Runx2* (Fig. 4D and E). Treatment of PDLSCs with 10 or 20 μM Y‐27632 significantly decreased the expression of *ALP* and *Runx2* at day 14 (*P* < 0.01). However, Y‐27632 had no significant effect on the expression of osteogenesis‐related gene (*P* > 0.05) and the expression of *Runx2* was slightly promoted by 10, 20 μM Y‐27632 compared with negative control group at day 7.

### Y‐27632 promoted formation of lipid droplets and adipogenic differentiation of PDLSCs

**Figure 5:**
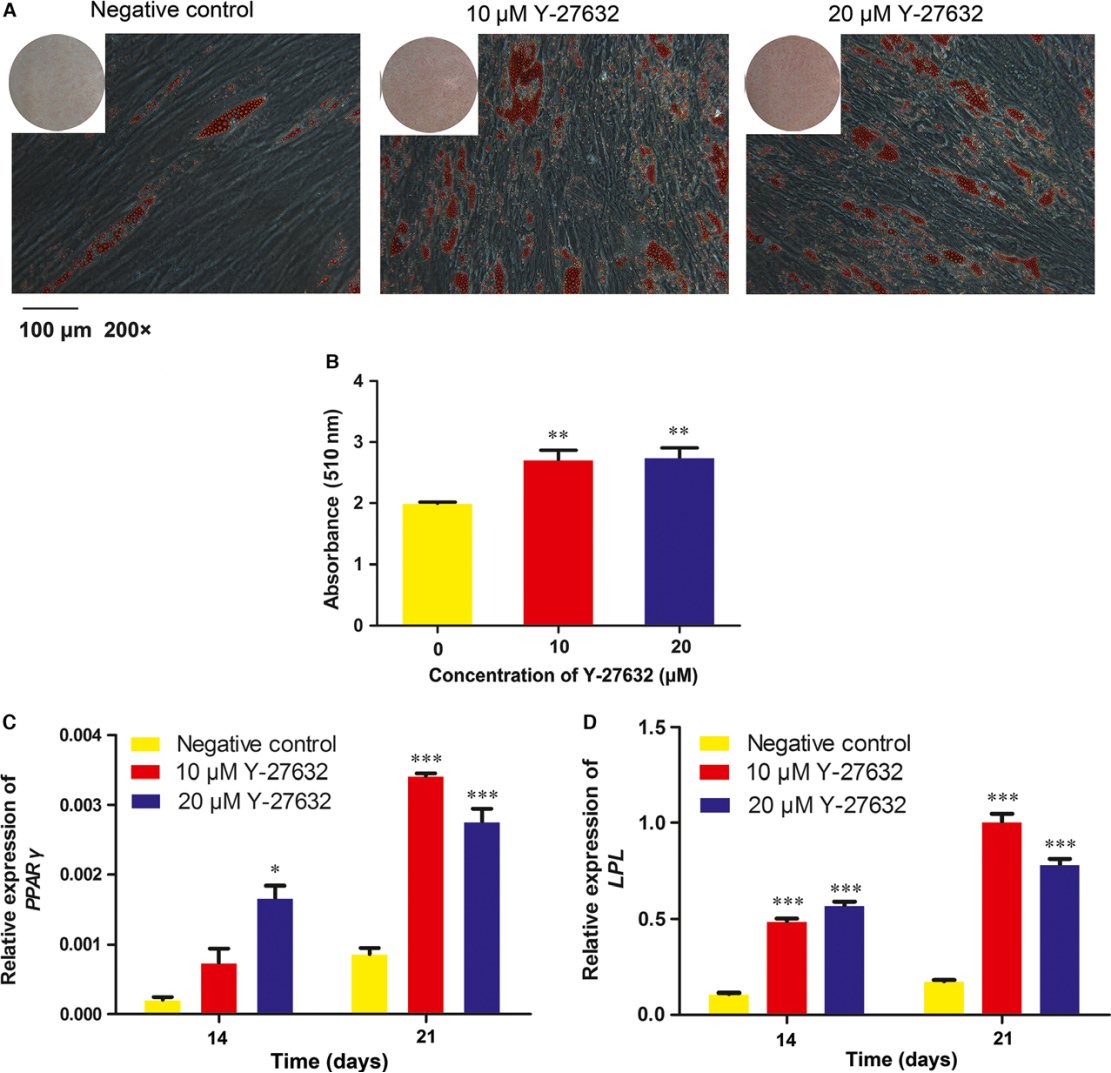
Effect of Y‐27632 on adipogenic differentiation. (**A**) PDLSCs were cultured in adipogenic medium for 28 days and Oil Red O staining was performed. scale bar:100 μm (×200). (**B**) Isopropyl alcohol was added and the amount of lipid droplets was quantified by absorbance at 510 nm. (**C, D**) PDLSCs were cultured in adipogenic medium for 14 and 21 days. The expression of adipogenesis‐related genes PPARγ and LPL were analysed by qRT‐PCR. Data were presented as the mean ± S.E.M. of three independent experiments. *P < 0.05;**P < 0.01;***P < 0.001 compared with negative control.

Lipid droplets formation was determined by Oil Red O staining (Fig. 5A) and the relative amount of lipid was quantified (Fig. 5B). Results showed that 10 and 20 μM Y‐27632 significantly promoted the formation of lipid droplets compared with control group (*P* < 0.01).

Moreover, the effect of Y‐27632 on adipogenic differentiation of PDLSCs was determined by evaluating the gene expression of *PPAR*γ and *LPL*. Treatment of PDLSCs with 10 or 20 μM Y‐27632 significantly increased the expression of *PPAR*γ and *LPL* in PDLSCs at day 14 and 21 (Fig. 5C and D).

### Y‐27632 maintained the stemness of PDLSCs

**Figure 6:**
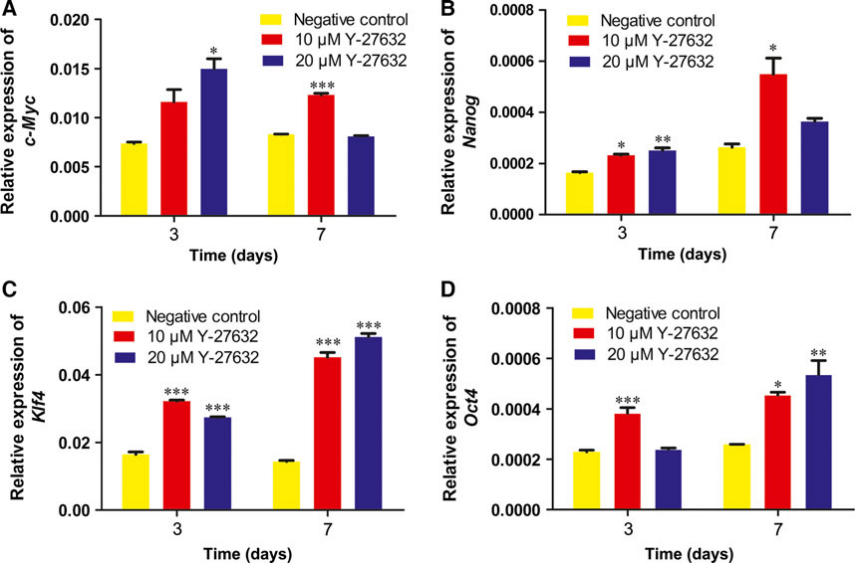
Y‐27632 up‐regulated the gene expression levels of c‐Myc, Nanog, Klf4 and Oct4 after 3, 7 days incubation. (A) Gene expression levels of c‐Myc after 3, 7 days of incubation. (B) Gene expression levels of Nanog after 3, 7 days of incubation. (C) Gene expression levels of Klf4 after 3, 7 days of incubation. (D) Gene expression levels of Oct4 after 3, 7 days of incubation. Data were presented as the mean ± S.E.M. of three independent experiments. *P < 0.05; **P < 0.01; ***P < 0.001 compared with negative control group.

To further define the stemness of PDLSCs after stimulation with Y‐27632, we analysed four stemness markers using qRT‐PCR. The gene expression levels of *c‐Myc, Nanog, Klf4* and *Oct4* with the treatment of Y‐27632 were significantly higher than that in control group at day 3 and 7 (*P* < 0.05; Fig. 6).

### ERK signal pathway involved in Y‐27632 induced PDLSCs proliferation

**Figure 7:**
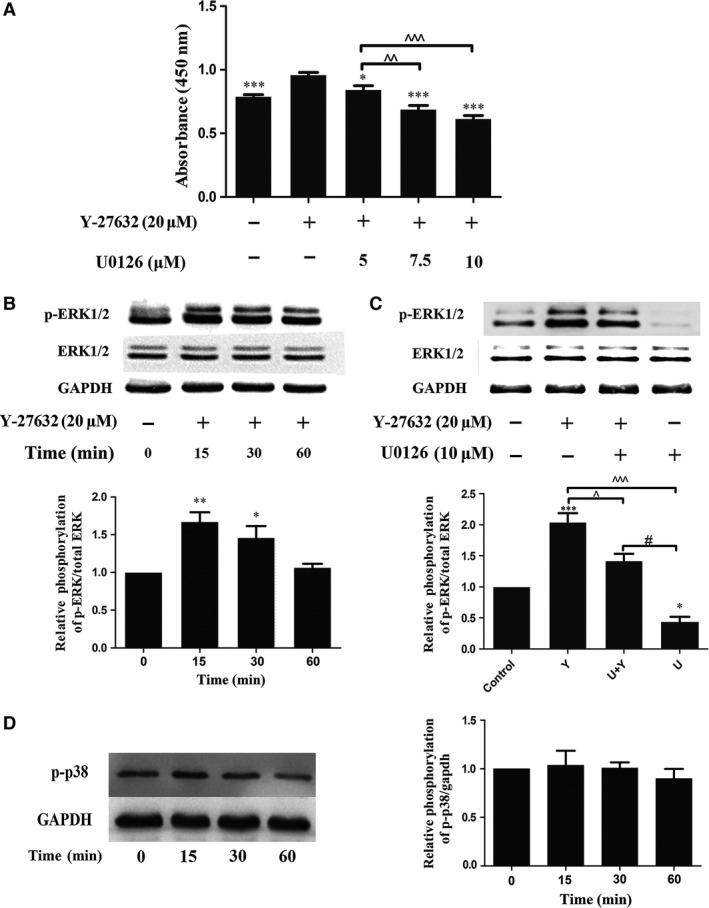
ERK signal pathway involved in Y‐27632 induced PDLSCs proliferation. (**A**) PDLSCs were treated with Y‐27632 (20 μM) and U0126 at 5, 7.5 and 10 μM for 48 hrs, and then 10 μl CCK8 solution was added to each well of the plate, and the absorbance was measured at 450 nm. *P < 0.05; **P < 0.01; ***P < 0.001 Y‐27632 alone treated group compared with other experimental groups and control group. ^^*P* < 0.01; ^^^*P* < 0.001 compared between two groups. (**B**) PDLSCs were treated with 20 μM Y‐27632 for 0, 15, 30 and 60 min., and the phosphorylation level of ERK was quantified by Western blot analysis. *P < 0.05; **P < 0.01 compared with control group. (**C**) To confirm that ERK inhibitor U0126 might attenuate Y‐27632‐induced ERK activity, p‐ERK was evaluated in Y‐27632‐treated PDLSCs with or without 10 μM U0126. *P < 0.05; ***P < 0.001 compared with control group. ^#*P* < 0.05; ^^^*P* < 0.001 compared with other experimental groups. (**D**)The phosphorylation of p38 was examined at 0, 15, 30, 60 min. Data were presented as the mean ± S.E.M. of three independent experiments.

PDLSCs proliferation was evaluated by CCK8 assay and EdU labelling assay. Y‐27632 enhanced cell proliferation in a dose‐dependent manner in the concentration range of 0–20 μM (Fig. 1A). However, Y‐27632‐induced growth of PDLSCs was significantly inhibited by the addition of ERK inhibitor U0126 at 5, 7.5 and 10 μM (Fig. 7A; *P* < 0.05). Afterwards, Western blot analysis was performed to explore the mechanism underneath. PDLSCs were stimulated with 20 μM Y‐27632 for 0, 15, 30, 60 min. The results indicated that Y‐27632 up‐regulated phosphorylation of ERK at 15 min. after stimulation, and then gradually decreased (Fig. 7B). Moreover, phosphorylation level of ERK decreased by the addition of U0126 at 10 μM (Fig. 7C). However, Y‐27632 had no effect on p38/MAPK phosphorylation (Fig. 7D; *P* > 0.05). These results indicated that ERK/MAPK rather than p38/MAPK signalling pathway involved in Y‐27632‐induced PDLSCs proliferation.

## Discussion

In this study, we demonstrated that Y‐27632 enhanced the proliferation and migration of PDLSCs *in vitro*. Moreover, Y‐27632 promoted wound‐healing ability, maintained the stemness, enhanced the formation of fat droplets and increased adipogenesis‐related gene expression levels while significantly inhibited ALP activity, the formation of mineral deposition and decreased osteogenesis‐related gene expression levels of human PDLSCs. Y‐27632 exhibited no significant effect on the apoptosis of PDLSCs. Furthermore, we demonstrated that the basic molecular mechanism for Y‐27632‐induced cell proliferation was ERK pathway.

Y‐27632 is an inhibitor of ROCK, which regulates a variety of cellular processes, including cellular growth, adhesion, migration, metabolism and apoptosis by controlling actin cytoskeleton assembly and cell contractions 19, 20. Previous studies have demonstrated that Y‐27632 is useful in the generation of human‐induced pluripotent stem cells by introducing reprogramming factors and can promote the induction efficiency of these stem cells 21, 22, 23. Furthermore, supplementary Y‐27632 can regulate the ability of stem cells to self‐renew and differentiate into derivatives of all three germ layers 24, 25, 26. More importantly, Y‐27632 was shown to greatly increase the long‐term proliferation of primary human keratinocytes, and unexpectedly, to enable these cells to efficiently bypass senescence and become immortal without a detectable cell crisis 27, 28, 29. Moreover, Y‐27632 could prevent mesenchymal stem cell (MSC) sheets from detaching and maintain the multilayered proliferation of confluent MSCs 30. In our study, 10 and 20 μM Y‐27632 promoted the proliferation and migration of PDLSCs while did not increase apoptosis at the same time. Previous studies demonstrated that Y‐27632 had anti‐apoptotic effect and inhibited the apoptosis of human keratinocytes 31, human corneal endothelial cells 32 and human embryonic stem cells 33. The inconsistency with our study may be attributed to the effect of Y‐27632 on apoptosis varying amongst different cell types.

Cell migration is indispensable in biological processes such as development, inflammation and wound healing. During repair and regeneration process, migration of stem cells is the key event. As the functional stem cells in periodontium, PDLSCs can migrate to the site of injury and differentiate into fibroblasts, osteoblasts and cementoblast and participate in periodontal regeneration. In earlier studies, dental pulp cells (DPCs) migration was increased by inhibition of ROCK 34, 35, and the findings differed from other studies in which ROCK inhibition reduced cell motility 36, 37. In our study, Y‐27632 supplementation promoted PDLSCs migration and accelerated cell migration over scratched wounds, which was in accordance with another study that Y‐27632 enhanced endothelial migration in a wound‐healing assay 38.

Pluripotency is the important characteristics for stem cells. In our study, the addition of Y‐27632 up‐regulated the gene expression levels of pluripotent markers of *Nanog, Oct4, c‐Myc* and *Klf4*. This was in accordance with the previous studies, wherein it was demonstrated that Y‐27632 increased *Oct‐3/4* expression in small colonies of human embryonic stem (hES) cells 39 and spatiotemporally altered the balance between pluripotency and early differentiation events 40.

Multipotential differentiation capacities are other characteristics of PDLSCs. Accumulating evidence suggests Rho‐ROCK signalling is involved as molecular switch for lineage‐specific cellular differentiation and Y‐27632 was found to promote differentiation of human embryonic stem cells into multiple cell types, including neurons, chondrocytes, osteocytes and smooth muscle cells 41. In our study, Y‐27632 increased fat lipid formation, up‐regulated the expression of adipogenesis‐related genes while inhibited ALP activity, decreased mineral nodule formation and discouraged osteogenesis‐related genes expression of human PDLSCs. These results suggested that the use of Y‐27632 might induce biased differentiation of PDLSCs and were corroborated by a previous study, which demonstrated that Y‐27632 down‐regulated PDL cell osteogenic differentiation and osteogenic gene expression 15. Conversely, it has been reported that treatment with Y‐27632 enhanced the osteoblastic differentiation of cultured murine neonatal calvarial cells, and increased the expression of BMP‐4 genes in ST2 cells. Moreover, the *in vivo s*tudy demonstrated that Y‐27632 in combination with the local delivery of rhBMP‐2 collagen composite enhanced ectopic bone formation 42. The possible mechanism underneath may be that Y‐27632 enhances cell migration to the defected site and promotes cell proliferation, and maintains pluripotency of stem/progenitor cells at the same time. Therefore, more stem cells are recruited to the defected site. At the later stage of wound healing, BMP‐2 promotes the osteogenic differentiation of stem cells and enhances bone regeneration. Therefore, the ROCK inhibitor such as Y‐27632 in conjunction with a local delivery of BMP‐2 may have therapeutic utility in the clinical setting. The strategy of pre‐treatment with Y‐27632 at early stage, and then application with BMP‐2 will synergistically enhances bone regeneration. Moreover, a recent study demonstrated that pre‐treatment with Y‐27632 markedly reduced LPS‐induced inflammatory response and oxidative stress and Y‐27632 might have a protective effect against LPS‐induced liver injury by improving mitochondrial function 43, which indicates that Y‐27632 may also act as a new strategy for treating inflammatory diseases. As is well known, periodontitis is a chronic inflammation of periodontium. Therefore, Y‐27632 may help to reduce periodontal inflammation and facilitate periodontal regeneration. Moreover, the application of Y‐27632 with ciliary neurotrophic factor (CNTF), a potent neurotrophic factor, resulted in additive effects on neurite outgrowth and regeneration 44.

Studies have found that ROCK and cell proliferation are linked by several common signalling pathways. Actually, the spatial and temporal regulation of activated ROCK leads to differential regulation of growth‐regulatory proteins in different cells 45 and the precise role of ROCK in PDLSCs growth and proliferation demands further investigations. In this study, we explored the signalling pathways to find the underlying mechanisms of Y27632‐induced cell proliferation. ERK and p38 are the members of mitogen‐activated protein kinases (MAPK) family. They are intracellular signalling pathways and play a pivotal role in many essential cellular processes, such as proliferation and differentiation 46. A major function of p38 and ERK is to transduce signalling of cell surface receptors to transcription factors in nucleus, which consequently triggers cellular events 47. In our study, we found that ERK, the most widely studied MAPK cascade, plays a crucial role in cell proliferation. CCK8 assay and EdU labelling assay showed that Y‐27632 enhanced cell proliferation in a dose‐dependent manner in the concentration range of 0–20 μM. Moreover, Y‐27632‐induced growth of PDLSCs was significantly inhibited by the addition of U0126, an inhibitor of ERK. Consistently, Western blot assay showed that inhibition of ROCK by Y‐27632 significantly increased the phosphorylation of ERK in cultured PDLSCs, and the effect was markedly attenuated by U0126 treatment. These results indicated that Y‐27632 could activate MAPK/ERK signalling which further promoted the proliferation of PDLSCs. Conversely, Y‐27632 had no effect on p38/MAPK phosphorylation, which indicated that ERK rather than p38/MAPK signalling pathway involved in Y‐27632‐induced proliferation of PDLSCs. It is reported that ERK also involves in cell migration 48. Taken together, the increase in cell proliferation, migration and wound‐healing capability may due to ROCK inhibition mediated by the ERK signalling pathway. The activation of ERK in PDLSCs may influence other biological activities including differentiation and apoptosis and the mechanism was not investigated in this study.

In summary, we demonstrated that ROCK inhibitor Y‐27632 could effectively increase the proliferation, migration, wound healing of PDLSCs, maintained the stemness and promoted adipogenic differentiation while inhibited osteogenic differentiation. The mechanisms may be mediated through the ERK signalling pathway. Understanding the cellular and molecular regulatory mechanisms of Y‐27632 may help to elucidate the role of ROCK inhibition in periodontal development and periodontal diseases. As Y‐27632 has already been employed clinically 49, this would indicate that it is safe for use in regenerative medicine.

## Conflict of interest

The authors confirm that there are no conflicts of interest.

## Supporting information


**Figure S1** Chemical structure of Y‐27632.
