# Supplementary figures and images for: Rho‐kinase inhibitor Y‐27632 facilitates the proliferation, migration and pluripotency of human periodontal ligament stem cells
Source: J Cell Mol Med. 2017 Jun 29;21(11):3100–12. doi: 10.1111/jcmm.13222 (PMC5661246; doi:10.1111/jcmm.13222)

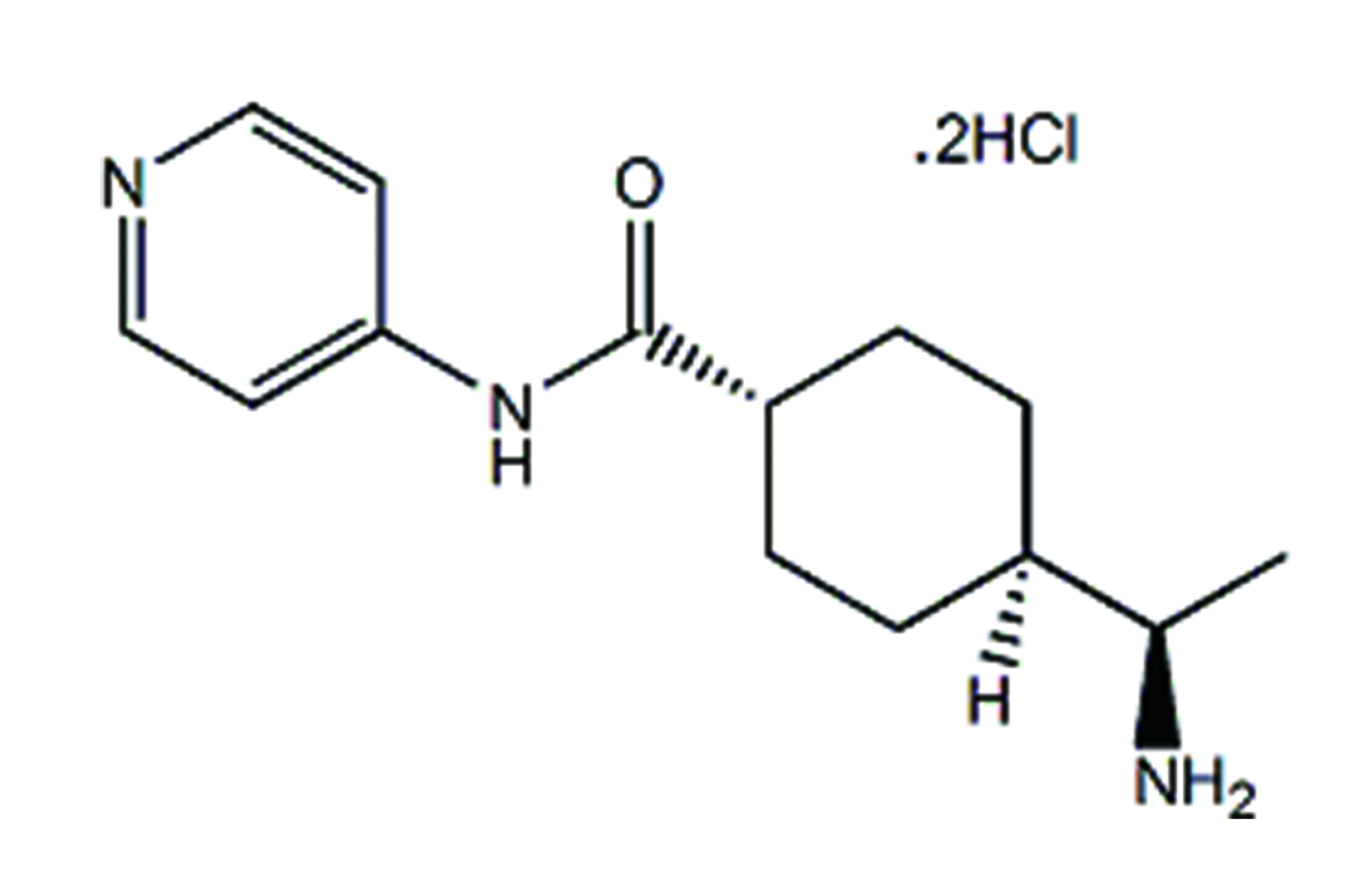

Supplement: Supplementary file 1 — Figure S1 Chemical structure of Y‐27632. [file JCMM-21-3100-s001.tiff]
